# Neural Crest Cell Survival Is Dependent on Rho Kinase and Is Required for Development of the Mid Face in Mouse Embryos

**DOI:** 10.1371/journal.pone.0037685

**Published:** 2012-05-21

**Authors:** Helen M. Phillips, Tania Papoutsi, Helena Soenen, Patricia Ybot-Gonzalez, Deborah J. Henderson, Bill Chaudhry

**Affiliations:** 1 Institute of Genetic Medicine, Newcastle University, Newcastle upon Tyne, United Kingdom; 2 Departamento de Pediatria, Hospital Infantil Virgen del Rocio, Seville, Spain; VIB & Katholieke Universiteit Leuven, Belgium

## Abstract

Neural crest cells (NCC) give rise to much of the tissue that forms the vertebrate head and face, including cartilage and bone, cranial ganglia and teeth. In this study we show that conditional expression of a dominant-negative (DN) form of Rho kinase (Rock) in mouse NCC results in severe hypoplasia of the frontonasal processes and first pharyngeal arch, ultimately resulting in reduction of the maxilla and nasal bones and severe craniofacial clefting affecting the nose, palate and lip. These defects resemble frontonasal dysplasia in humans. Disruption of the actin cytoskeleton, which leads to abnormalities in cell-matrix attachment, is seen in the *RockDN;Wnt1-cre* mutant embryos. This leads to elevated cell death, resulting in NCC deficiency and hypoplastic NCC-derived craniofacial structures. Rock is thus essential for survival of NCC that form the craniofacial region. We propose that reduced NCC numbers in the frontonasal processes and first pharyngeal arch, resulting from exacerbated cell death, may be the common mechanism underlying frontonasal dysplasia.

## Introduction

The vertebrate face is largely formed from neural crest-derived mesenchyme, covered in ectoderm, with a small endodermal contribution (reviewed in [1]). Although the surface ectoderm and pharyngeal endoderm have important signalling roles in the patterning of the forming craniofacial region, it is the cranial neural crest cells (NCC) that form most of the bone and cartilage within the head, the teeth and the cranial ganglia. The rostral NCC make extensive contributions to the frontonasal skeleton and the calvaria of the skull, whereas the more caudal cranial NCC migrate into the pharyngeal arches where they form the mandible and maxilla, the middle ear and the hyoid bone (reviewed in [2]]. The forming craniofacial region is initially defined by a series of swellings or prominences. The medial frontonasal prominence forms the forehead, middle of the nose, philtrum of the upper lip and primary palate. Three paired prominences, derived from the first pharyngeal arch, form the lateral regions of the mid and lower face, associated with the maxilla, the mandible and the secondary palate [1]. In order for these initially featureless prominences to give rise to their complex derivatives, processes of growth, expansion and fusion have to be orchestrated. Interactions between NCC, the surface ectoderm, mesoderm and endoderm are all crucial for the normal development of the developing face (reviewed in [3]]. Studies in animal models, particularly mouse and chick, have highlighted the importance of specific signalling cascades, including the sonic hedgehog, fibroblast growth factor and bone morphogenetic protein pathways, in the development of the craniofacial region [1], [2].

The two Rho kinase isoforms (Rock 1 and 2) are highly conserved serine/threonine kinases that play essential roles in fundamental cellular processes such as contraction, adhesion, migration, apoptosis and proliferation. Almost all of the analyses of Rock function have been carried out in cell culture. In these *in vitro* experiments, within the two-dimensional environment of the culture dish, Rock is found at the rear of migrating cells and regulates stress fibre and focal adhesion assembly, facilitating cell body and trailing edge retraction [4], [5]. *Rock1* and *Rock2* show similar expression patterns in the developing embryo [6] and both the *Rock1* and *Rock2* single knockout mice exhibit omphalocele and open eyelids at birth [7], [8], whereas only the *Rock2* null mice develop defects in development of the placenta and intra-uterine growth retardation [7], suggesting that the two genes work co-operatively in the development of these structures and have at least some non-redundant functions. The data showing that *Rock1/Rock2* double heterozygotes have the same embryonic phenotype as do the single homozygotes for *Rock1* or *Rock2* on some genetic backgrounds [7]–[9], suggests that the overall level of Rock protein (Rock1 or Rock2) may be critical. *Rock1/Rock2* double null mice have not been described in the literature. However, chemical inhibition of Rock (using Y27632) in neurulating chicken and mouse embryos results in a variety of malformations, including brain abnormalities and neural tube defects [6]. The broad and overlapping expression of *Rock1* and *Rock2* in the developing embryo, together with the normal development of the majority of the embryo in the single knockouts for *Rock1* or *Rock2* [7]–[9] suggests there may be some degree of functional redundancy. Further studies have implicated Rock in delamination of NCC in quail embryos [10] and in NCC migration in Xenopus and zebrafish [11] although this has not been demonstrated in mammalian embryos.

In this study we utilised transgenic mice that allowed *cre*-mediated tissue-specific expression of dominant-negative (DN) Rock (*RockDN*); [12] to explore its role in NCC using *Wnt1-cre* [13] mice. In the *RockDN* mice, the Rho kinase RB/BH (TT) fragment [14] is expressed under the control of the cytomegalovirus promoter specifically blocking the function of both Rock1 and Rock2 isoforms. The point mutations introduced into the Rock RB/BH (TT) interact with endogenous Rock and abolish Rho binding activity, but have no effect on the activity of related kinases [15]. Thus, expression of Rock RB/BH (TT) can be used to specifically block Rho kinase function in vivo. Use of Cre-loxP technology means that the RockDN protein can be expressed in the cell type of choice. Thus the use of these mice can bypass any early lethality that might result from the loss of both Rock isoforms during early development, but also overcome potential functional redundancy. The *RockDN;Wnt1-cre* embryos developed severe NCC-related defects affecting the craniofacial region including hypoplasia of structures derived from the frontonasal prominence and first pharyngeal arch.

## Results

### Specific knock-down of Rock in NCC causes severe craniofacial malformation

**Figure 1:**
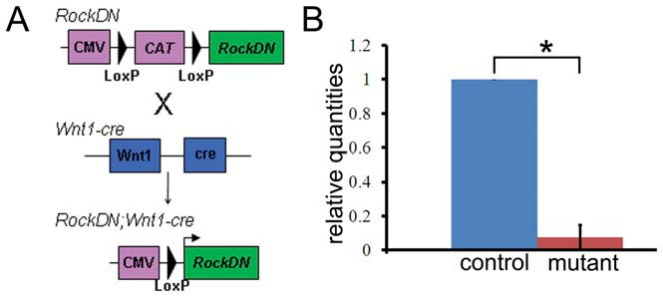
Conditional expression of *RockDN* in NCC. **A**) Strategy for expressing *RockDN* construct in NCC. **B**) Quantitative real time PCR for the *CAT* gene cassette, using RNA extracted from pharyngeal arches 1 and 2, from E11.5 mutant *RockDN^+^;Wnt1-cre^+^* and control embryos. There is a statistically significant (P<0.05 *), 13 fold decrease, in the expression of the *CAT* box in the mutant sample, as calculated using the one-way Anova test.

The role of Rock isoforms in migrating NCC, and their derivatives, was investigated using mice expressing a *RockDN* construct [12] under the control of the *Wnt1-cre* driver [13], Figure 1A. The RockDN protein RB/PH (TT) [12], [14] is unable to bind to Rho and specifically blocks Rock function [15]. Female mice heterozygous for the *RockDN* construct and male mice heterozygous for the *Wnt1-cre* construct were mated (Figure 1A), with resulting litters containing embryos of the genotypes: *RockDN^+^;Wnt1-cre^+^* (hereafter referred to as *RockDN*), *RockDN^+^;Wnt1-cre^−^*, *RockDN^−^;Wnt1-cre^+^* and *RockDN^−^;Wnt1-cre^−^*; the latter three genotypes were phenotypically indistinguishable and acted as controls. To assess the efficiency of *cre* recombination we evaluated the levels of the *CAT* gene cassette transcripts in pharyngeal arches 1 and 2 of embryonic heads taken from *RockDN^+^;Wnt1-cre^+^* mutants and *RockDN^+^;Wnt1-cre^−^* controls at E11.5, as this tissue was rich in NCC (Figure S1 C,D). Quantitative real time PCR revealed a significant 13 fold reduction in levels of the *CAT* cassette in the mutant heads (P = 0.006), compared with transgenic embryos where *cre* was not present; a reduction to almost undetectable levels (Figure 1B). Efficient removal of the *CAT* cassette is consistent with expression of dominant-negative Rock in the majority of NCC. Notably, expression of RockDN in all cells of the embryo, by intercrossing *RockDN* mice with *PGK-cre* mice [16] suggested that ubiquitously blocking Rock function results in embryonic death before E9.5 (data not shown).

**Figure 2:**
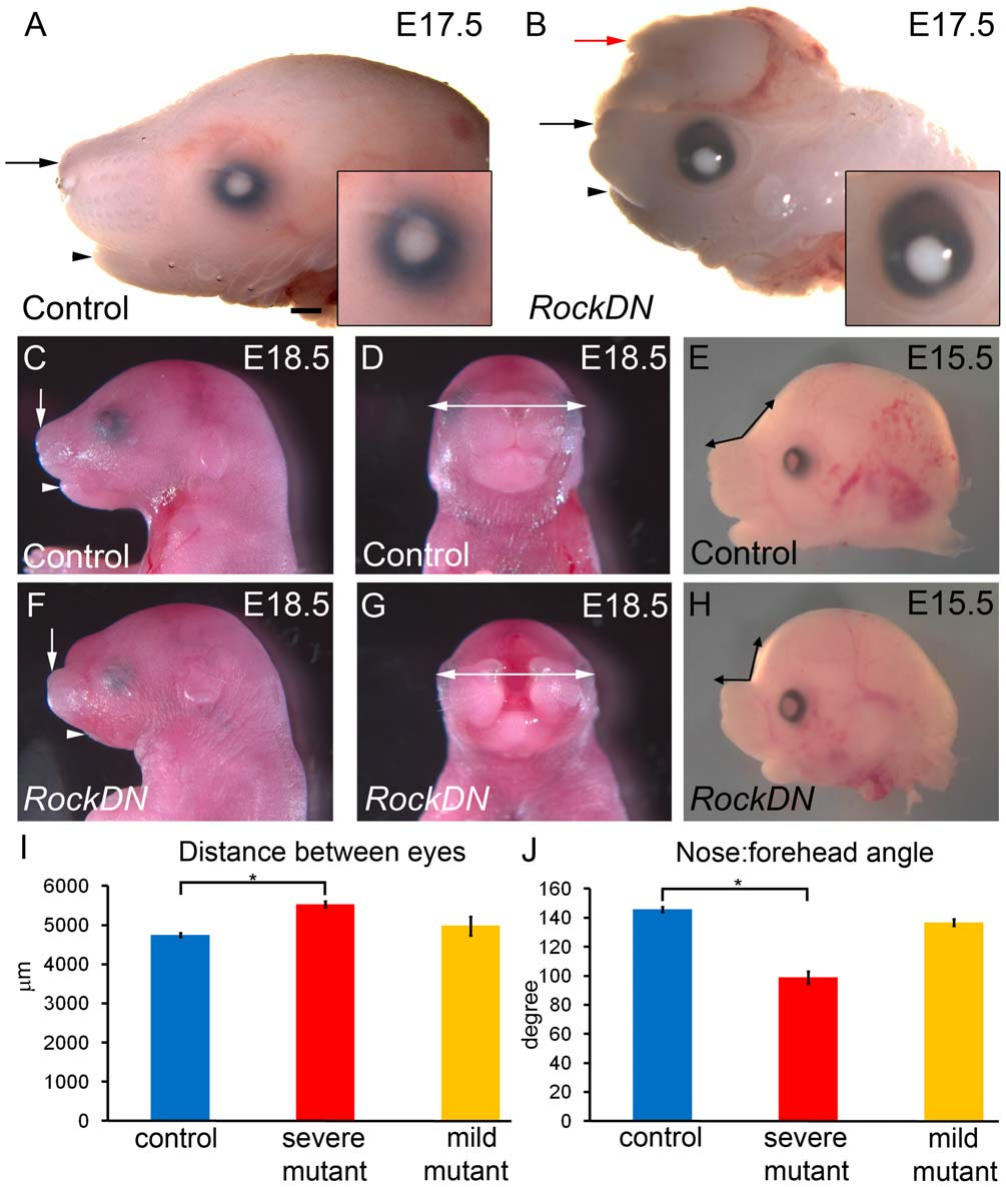
Craniofacial malformations in *RockDN;Wnt1-cre* embryos. **A,B**) Severe frontonasal (arrow), maxilla and mandible hypoplasia (arrowhead) and exencephaly (red arrow) in E17.5 *RockDN;Wnt1-cre* embryo (B), compared with control littermate (A). Note also the abnormally shaped eye and absence of eyelids in the mutant embryo (magnified inserts in A and B). **C,F**) Lateral views show the abnormal development of the snout in mildly affected *RockDN;Wnt1-cre* embryos at E18.5. Mild truncations of the frontonasal processes (arrow) and mandible (arrowhead) are apparent. **D,G**) Clefting of the upper lip and nose are apparent in a frontal view of an E18.5 *RockDN;Wnt1-cre* embryo (G; same fetus as in F). Double arrows are the same length in D and G highlighting the increased intra-ocular distance in *RockDN;Wnt1-cre* embryos. **E,H,I,J**) Measurements were taken from E15.5 heads (11 control embryos, 6 severely affected *RockDN;Wnt1-cre* mutants and 3 mildly affected *RockDN;Wnt1-cre* mutants). The distance between the eyes and the size of the angle between the nose and the forehead was measured (double arrow in E,H) and the results are shown in graphs I and J, respectively. In both cases, the measurements in the severely affected mutant embryos were significantly different from the controls (P<0.0001 *). There were no significant differences between controls and the mildly affected mutants for either measurement. Scale bar in A–D,F,G = 500 µm; E,H = 375 µm.

We reviewed craniofacial development in the *RockDN;Wnt1-cre* embryos. By late gestation, *RockDN* embryos showed craniofacial malformations affecting the fronto-medial aspect of the head, although these varied in severity. The predominant feature was mid-facial clefting in combination with hypoplasia of the frontonasal region (Figure 2). Those with the severest phenotype (47/54, from E11.5 to E18.5) had marked reductions of the frontonasal region, maxilla and mandible (Figure 2 A,B). Facial clefting was apparent in 50/54 embryos (Figure 2G and Figure S1 C–H), although in three cases this was apparent externally only as a bifid nose (Figure S1J). At E15.5, the distance between the eyes and the width of the frontonasal processes was larger in mutant embryos compared to control littermates (P = 4.202×10^−6^, Figure 2I and data not shown), consistent with hypertelorism and broader skulls in the mutants. There was no significant difference between the heights of the heads (data not shown). The snout was also upturned in the *RockDN* embryos at E15.5, shown by a decreased angle between the snout and the forehead when compared to control littermates (P = 3.208×10^−8^; Figure 2 E,H,J). There was no significant difference between the two groups for any measurement at E11.5, despite mid-facial clefting being apparent at that stage (data not shown).

**Figure 3:**
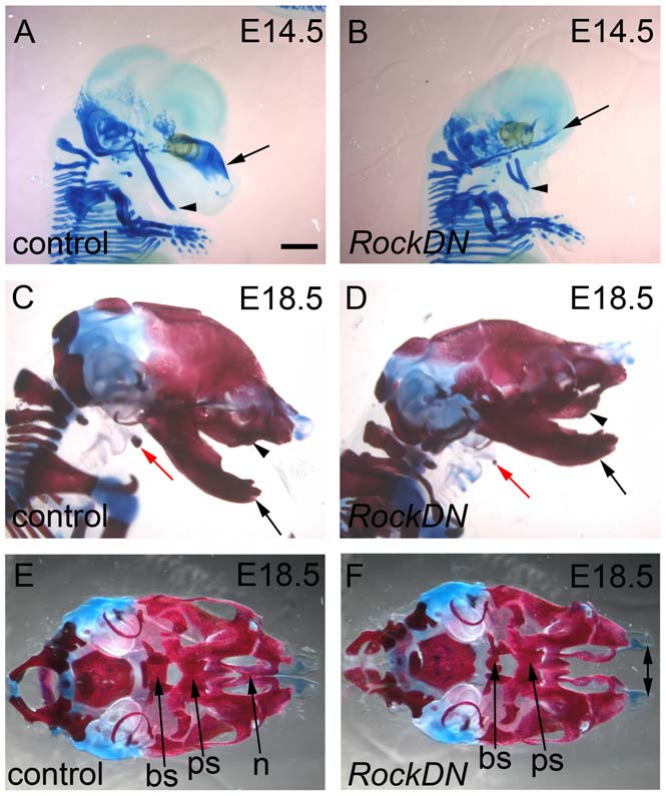
Abnormalities in formation of the craniofacial bones in *RockDN;Wnt1-cre* embryos. **A,B**) In severely affected *RockDN;Wnt1-cre* embryos at E14.5, the frontonasal bones (stained with alcian blue) are absent (arrow in B, compare to A). Meckel's cartilage is also reduced in size (arrowhead in B, compare with A). **C,D**) Bone (red) and cartilage (blue) staining of a mildly affected *RockDN;Wnt1-cre* embryo at E18.5 (D), shows that the maxilla (arrowhead) and mandibular (arrow) bones are well formed, although the hyoid bone (red arrow) is reduced in size in mutant embryos. **E,F**) Inferior views of the base of the skull in mildly affected embryos shows that the basisphenoid and the presphenoid bones are hypoplastic in *RockDN;Wnt1-cre* embryos, whereas the nasal septum is completely missing. Moreover, the maxillary bones are widely separated in mutant embryos (double arrow in F), compared to control littermates (E). bs = basisphenoid; n = nasal septum; ps = presphenoid. Scale bar = 500 µm.

At E17.5–E18.5, two out of four *RockDN* embryos had failed to form eyelids (Figure 2B), as seen in mice null for either *Rock1* or *Rock2* [7]–[9]. This was not unexpected, as the eyelids form from NCC-derived mesenchyme [17]. Some abnormalities may have occurred as secondary events. The hypertelorism observed throughout gestation is likely to be secondary to the mid-facial clefting as this produces a broader skull. In addition, two out of four *RockDN* embryos examined at E17.5–E18.5 had exencephaly (Figure 2B). As all embryos collected earlier in gestation had closed cranial neural tubes, this does not suggest a primary involvement of *Rock* in neural tube closure, but rather suggests secondary opening, likely as a consequence of a defect in the NCC-derived calvarias bones. Cartilage staining of E14.5 embryos revealed that the nasal capsular cartilage is missing and the Meckel's cartilage appears shortened in the *RockDN* embryos (Figure 3 A,B). Bone and cartilage staining of more mildly affected E18.5 embryos demonstrated that the majority of the cranium was well formed although there were subtle abnormalities in some NCC-derived bones (Figure 3 C–F); specifically there was mis-shaping and hypoplasia of the basisphenoid bone (Figure 3 E,F). The bones of the nasal septum and the maxillary bones failed to meet in the midline (double arrow in Figure 3F) and the hyoid bone was hypoplastic (red arrow in Figure 3 C,D). Thus, expression of the *RockDN* construct in NCC resulted in defects in the craniofacial structures derived from the frontonasal prominence and the first pharyngeal arches. Severe abnormalities of the outflow region of the heart were also observed in all *RockDN;Wnt1-cre* embryos by E11.5; these are described elsewhere (Phillips *et al.*, manuscript in preparation).

### Expression of Rock1 and Rock2 in developing craniofacial structures

**Figure 4:**
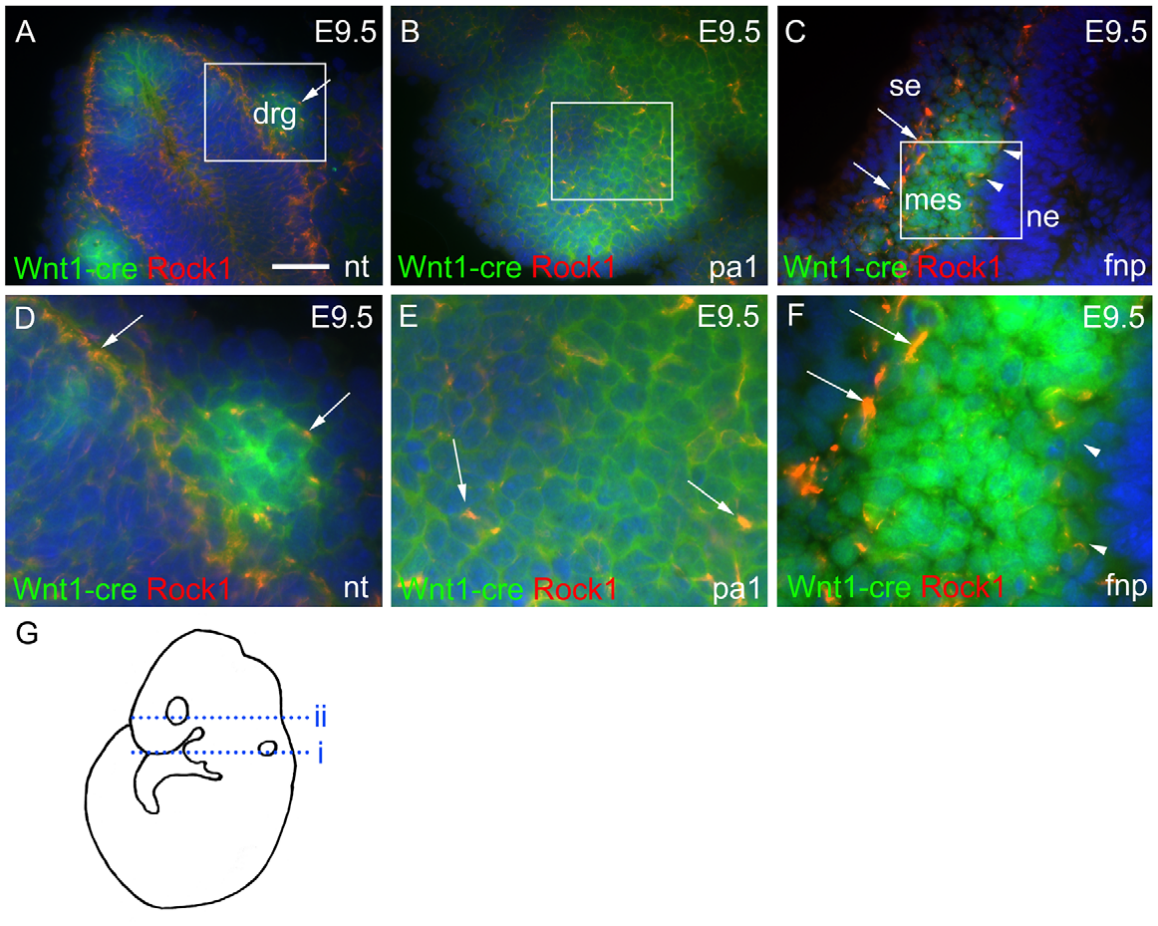
Expression of Rock1 in the developing head. **A–F**) D–F are magnified regions from A–C, respectively. At E9.5, Rock1 protein (red) is expressed in the neural tube and the dorsal root ganglia, where it colocalises with Wnt1-cre+ve NCC (green, detected by GFP antibody, arrows in A,D), and in the pharyngeal tissue (B,E). In the frontonasal processes (C,F) Rock1 is expressed at the boarders of NCC rich ectomesenchyme and the surface ectoderm (arrows) and neural ectoderm (arrowheads), which are devoid of NCC. **G**) The position of the transverse sections shown in A–C are illustrated on a cartoon of an E9.5 embryo. Line i represents the position through the neural tube (A) and pharyngeal arch 1 (B) and line ii is the level of the fronotnasal processes (C). drg = dorsal root ganglia; fnp = frontonasal processes; mes = mesenchyme; ne = neural ectoderm; nt = neural tube; pa1 = pharyngeal arch 1; se = surface ectoderm. Scale bar in A–C = 45 µm.

Inter-crossing of *ROSA-EYFP* mice with *Wnt1-cre* enabled the co-localisation of Rock1 protein with NCC. At E9.5 Rock1 was expressed in the neural tube and the dorsal root ganglia (Figure 4 A,D). Expression localised to the perimeter of the cells was found in the mass of the NCC within the pharyngeal arches (Figure 4 B,E and Figure S2 A–D) and in the frontonasal processes (Figure 4 C,F). Rock1 immunoreactivity was most abundant, however, at the boundaries of the NCC-derived mesenchyme with the epithelia of the surface ectoderm and the neural ectoderm in the frontonasal processes (arrows and arrowheads in Figure 4 C,F). Rock2 was expressed in similar NCC-rich regions (Figure S2 E–G) confirming that both isoforms were found in regions rich in migrating and post-migratory NCC. By E11.5, both Rock1 and Rock2 proteins were down-regulated in the craniofacial region (data not shown).

### Reduction of NCC corresponds to defects in cranial NCC derivatives

**Figure 5:**
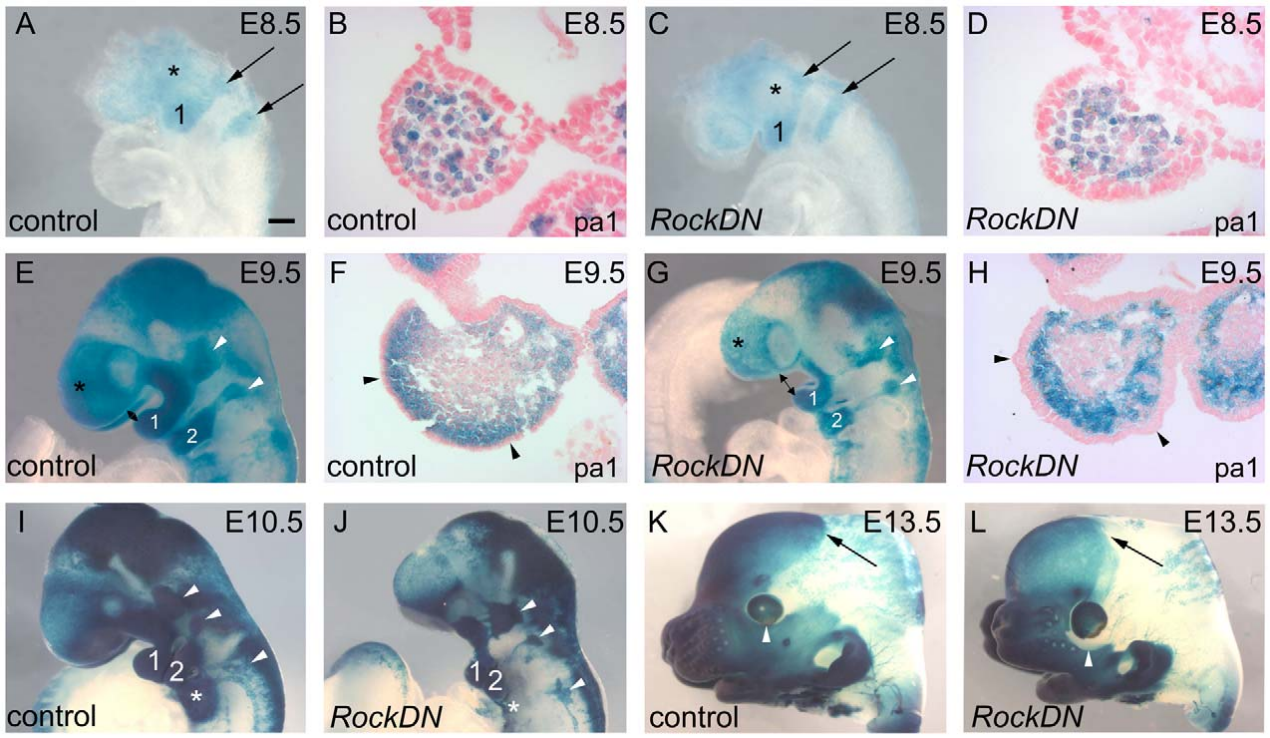
β-gal staining of NCC (blue) in *RockDN;Wnt1-cre* embryos. **A–D**) At E8.5, the numbers of NCCs migrating towards pharyngeal arches 1 and 2 (arrows in A,C) and within pharyngeal arch 1 (B and D) are similar. There is a reduction in NCCs populating the region anterior to the developing eye (asterisks in A,C). **E–H**) At E9.5, there is increased distance between the frontonasal regions and the first pharyngeal arch (double arrows in E,G). There is a reduction in the numbers of NCC in the head (asterisks in G, compare to E) and the first pharyngeal arch (F,H). The surface ectoderm (arrowheads in F,H) is thickened and uneven in the mutant (compare H with F). **I,J**) At E10.5, the hypoplasia of the frontal region is more obvious in severely affected *RockDN;Wnt1-cre* embryos and the pharyngeal arches are frequently hypoplastic (numbered 1, 2 and * (posterior pharyngeal arches 3–6)). The cranial ganglia are misshapen and smaller in size in *RockDN;Wnt1-cre* embryos (white arrowheads in E,G,I,J). **K,L**) By E13.5, abnormalities in the distribution of NCC are seen even in the more mildly affected embryos, with reduced β-gal staining observed in the midline of the forming calvarias bones (arrows) and below the eye (white arrowheads). The frontonasal region is mildly truncated. pa1 = pharyngeal arch 1; 1 = pharyngeal arch 1; 2 = pharyngeal arch 2. Scale bar in A,C = 200 µm; B,D = 2 µm; E,G = 160 µm; F,H = 40 µm; I–L = 600 µm.

We used the *Rosa 26R* (β-galactosidase; β-gal) reporter to investigate the extent of migration of NCC and the distribution of their derivatives with regard to the observed craniofacial phenotypes. At E8.5, control and *RockDN* embryos were indistinguishable based on their external appearance. Staining for β-gal showed normal migration of NCC towards pharyngeal arches 1 and 2 (Figure 5 A,C), suggesting that NCC induction and delamination had occurred normally. However, there was a reduction in β-gal expression anterior to the stream of NCC migrating to the 1^st^ pharyngeal arch (Figure 5 A,C). At E9.5–E10.5, the mutant embryos were still not reliably distinguishable from control littermates on the basis of their craniofacial phenotype. However, the β-gal expression was generally more patchy in the craniofacial region of *RockDN* embryos examined at E9.5–E10.5 (Figure S1 A,B and Figure S3) and there appeared to be fewer β-gal-stained NCC in all embryos examined at this stage (9/9). Although the frontonasal structures appeared hypoplastic in 87% of *RockDN* mutant embryos by E11.5–E18.5 (Figure S1 C–H), β-gal expression was confluent in the developing facial prominences. It was deficient, however, in the posterior parts of the medial calvarias bones in embryos with the most severe craniofacial phenotype (data not shown) and below the eyes (Figure 5 K,L). In addition to the structural abnormalities already described, the thymus was ectopically located in the cervical region (data not shown) and the cranial ganglia were misshapen and reduced in size (arrowheads in Figure 5 E,G,I,J and data not shown).

The β-gal staining pattern also revealed abnormalities in NCC distribution during the development of the pharyngeal arches. At E8.5, the streams of NCC migrating towards the pharyngeal arches were similar in both wild type and *RockDN* mutant embryos (arrows in Figure 5 A,C). However, at E9.5, and persisting at E10.5, the first and second pharyngeal arches were hypoplastic and the β-gal expression in the arches was less intense and patchy (Figure 5 E–J). These data suggest that although the formation, delamination and early migration of NCC appeared to occur normally at E8.5 in the *RockDN* mutants, NCC numbers declined later in development.

### Ectopic and exacerbated apoptosis in *RockDN* NCC

**Figure 6:**
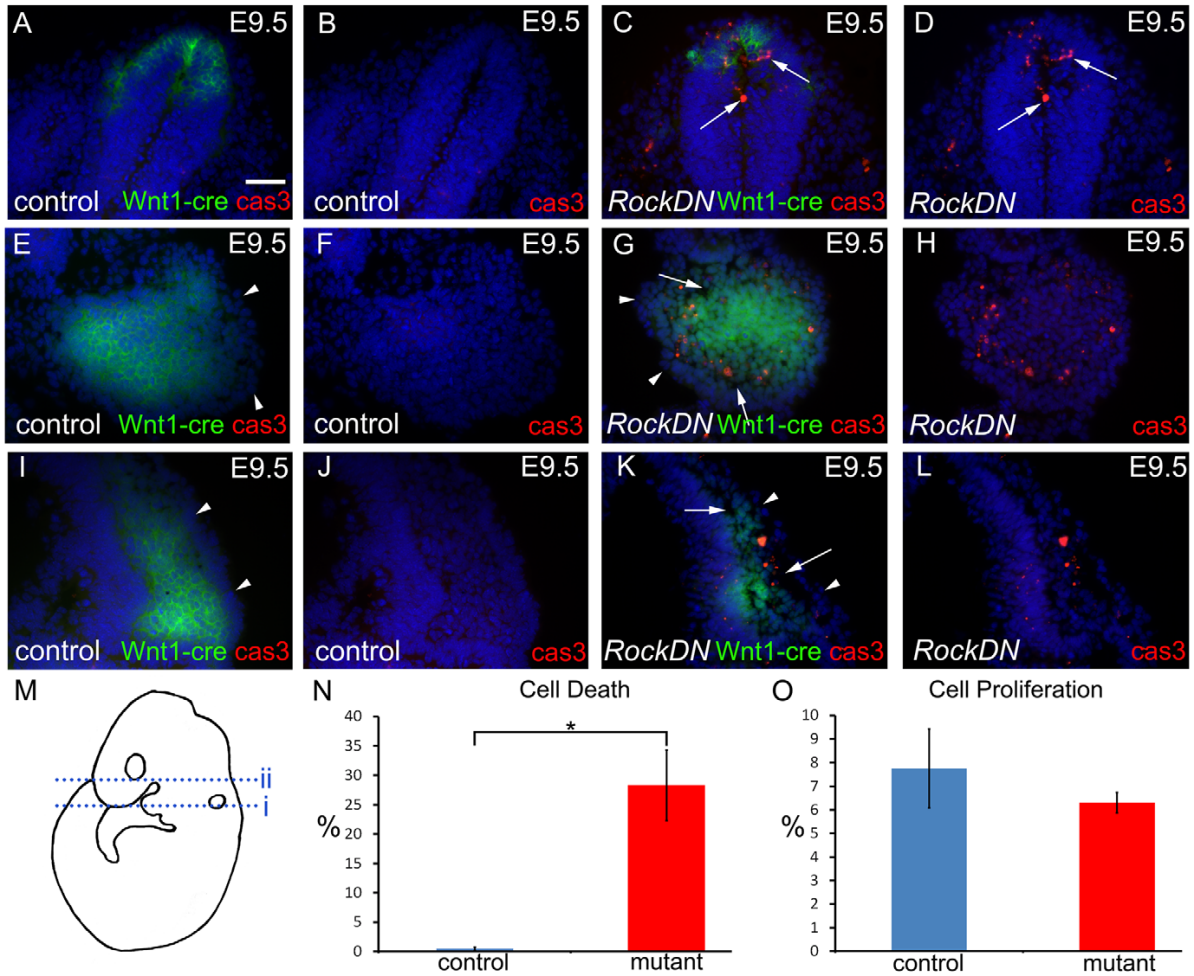
Ectopic and excessive cell death in E9.5 *RockDN;Wnt1-cre* embryos. A–D are sections through the neural tube (line i in M), E–H through the pharyngeal arch 1 (line i in M) and I–L through the frontonasal processes (line ii in M). A,C,E,G,I,K show caspase 3-expressing cells (red) and Wnt1-cre+ve NCCs (green). B,D,F,H,J,L are the same sections but only showing the caspase 3-expressing cells. **A–D**) Whereas only very occasional activated caspase 3-expressing, dying, cells (red) are seen in the neural epithelium in control embryos, there are many dying cells observed in the dorsal part of the neural tube, from which the NCC emerge, in *RockDN;Wnt1-cre* mutant embryos (arrows in C and D). **E–L**) Very few activated caspase3-expressing cells are observed in the NCC-derived ectomesenchyme of pharyngeal arch 1 (E,F) and the frontonasal region (I,J) in control embryos. In contrast, many dying cells are seen in corresponding regions from *RockDN;Wnt1-cre* mutants (G,H,K,L). The surface ectoderm in the mutant is more irregular, compared to controls (arrowheads in E,G,I,K) and the inner NCC-derived ectomesenchyme is loosely arranged with gaps between the cells (arrows in G,K). **N,O**) The mean apoptotic and mitotic indexes were calculated for NCC within E9.5 pharyngeal arches. There is a significant increase in cell death in the mutant samples compared to controls (P = 0.019; * in N). There is no significant difference in cell proliferation between the two samples (P = 0.433; O). Scale bar = 50 µm.

Reduced Rock function is associated with increased apoptosis in developing motor neurones [12]. We therefore examined the expression of activated caspase 3 in the developing *RockDN* embryos, hypothesising that increased levels of apoptosis in the affected tissues might explain the emergent phenotype. NCC migration begins at E8.0 in the cranial region of mouse embryos and is complete by the end of E9.5 [18]. At E8.5, after the first NCC had delaminated from the neural tube, but before the appearance of any craniofacial phenotype, there was little if any activated caspase 3 labelling anywhere in the control embryos. However, a few activated caspase 3-expressing, dying, cells were observed in the frontonasal region and pharyngeal arches of *RockDN* embryos at this stage (data not shown). By E9.5, and continuing at E10.5, activated caspase 3-positive cells were abundant in the dorsal part of the neural tube in *RockDN* embryos, localising to the *Wnt1-cre* domain (Figure 6 A–D). This correlates with the latter stages of NCC delamination from this region but also continues after migration from the cranial neural tube is complete. The mesenchymal cells within pharyngeal arches 1–3 were compact in control embryos by E9.5. The surface ectoderm formed a smooth layer surrounding the inner ectomesenchyme (Figures 5F and 6 E,F). This outer layer appeared uneven in the *RockDN* mutants and the mesenchymal cells appeared loosely arranged and disorganised (Figures 5H and 6 G,H). Similar observations were made in the frontonasal processes of control and stage matched mutant embryos (arrowheads in Figure 6 I–L). Examination of the craniofacial region of *RockDN* embryos revealed that more than 25% of NCC were caspase 3-positive in pharyngeal arches 1–3 and the dorsal frontonasal prominences at E9.5 and E10.5 (Figure 6 E–N and data not shown), compared with only occasional caspase 3-expressing cells in comparable sections from control littermates (P = 0.019; Figure 6 E–N and data not shown). Thus, this high incidence of cell death likely explains the less compact appearance of the ectomesenchyme of the pharyngeal arches and frontonasal processes. In contrast to the elevated cell death, analysis of cell proliferation in NCC-rich regions of the developing craniofacial region revealed no significant differences between *RockDN* and control littermates at any stage examined (P = 0.433; Figure 6O and data not shown). These data suggest that the progressive reductions in NCC numbers observed in the craniofacial and pharyngeal regions at E9.5–E10.5, and the hypoplasia of NCC-derived structures observed in these regions later in development, were caused by loss of NCC by apoptosis.

### Abnormal NCC-matrix interactions are associated with NCC death in craniofacial tissues of *RockDN;Wnt1-cre* embryos

**Figure 7:**
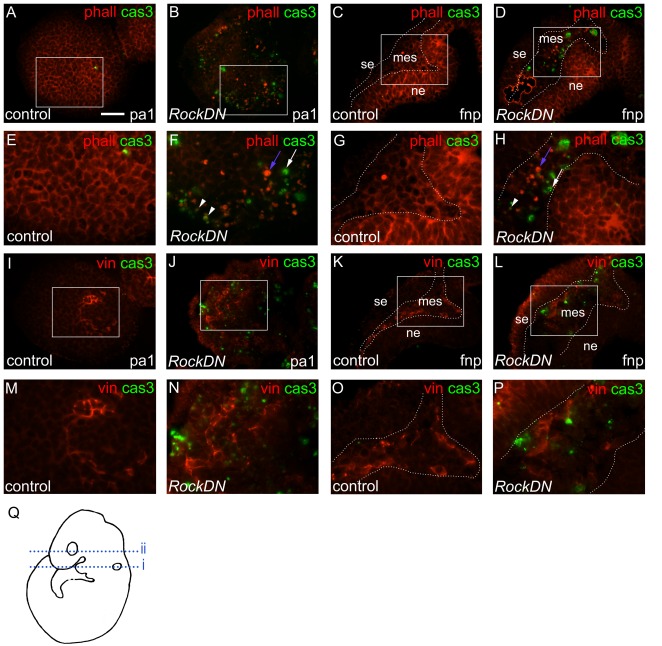
Disruption of the actin cytoskeleton and vinculin-containing focal contacts in E9.5 *RockDN;Wnt1-cre* embryos. A,B,E,F,I,J,M,N (line i in Q) are sections from first pharyngeal arch and C,D,G,H,K,L,O,P (line ii in Q) are from the frontonasal processes. A–H show phalloidin (red) and caspase 3 (green) immunofluorescence, with E–H being magnified regions as shown by the boxes in A–D, respectively. I–L show vinculin (red) and caspase 3 (green) dual immunofluorescence, with M–P being magnified regions as shown by boxes in I–L. The dotted lines in C,D,G,H,K,L,O,P indicate the boundary between the inner ectomesenchyme and the neural ectoderm and the surface ectoderm. **A–H**) Filamentous actin, labelled with phalloidin (red) outlines the cells in NCC-derived ectomesenchyme and neural ectoderm in the first pharyngeal arch (A,E) and frontonasal processes (C,G) of control embryos. Cortical phalloidin staining is lost in the ectomesenchyme from *RockDN;Wnt1-cre* mutants (F,H) but is maintained in the neural ectoderm (compare G with H). In addition, intense phalloidin-labelled foci are observed throughout the ectomesenchyme of the *RockDN;Wnt1-cre* mutants (dense red foci, blue arrow in F,H). Green caspase 3-positive cells are interspersed (white arrow) and overlapping with the phalloidin-intense cells (arrowheads in F,H). **I–L**) Vinculin and caspase 3 staining. In the pharyngeal arch the vinculin staining is not restricted to the centre of the arch in the mutant (compare J with I). In the frontonasal processes vinculin outlines the boundary between the surface ectoderm and the neural ectoderm with the inner NCC-derived ectomesenchyme (dotted lines in K,L). This discrete vinculin staining is lost in the *RockDN;Wnt1-cre* mutants (compare P with O). cas3 = activated caspase-3; mes = mesenchyme; ne = neural ectoderm; phall = phalloidin; se = surface ectoderm; vin = vinculin. Scale bar = 50 µm.

As Rock is a key regulator of the actin cytoskeleton, and disruption of the actin cytoskeleton has been linked to induction of cell death in a variety of contexts [19] we carried out phalloidin staining for filamentous actin in sections taken from E9.5 control and *RockDN* embryos, as this was the stage when cell death was at its peak. In control embryos, filamentous actin was found in a marked cortical distribution, lining the perimeter of the cell, in the NCC-derived ectomesenchyme of the pharyngeal arches (Figure 7 A,E) and frontonasal region (Figure 7 C,G), as well as the non-NCC-derived neural ectoderm of these regions. Cortical phalloidin staining was markedly reduced in the mesenchyme of the pharyngeal arches (Figure 7 B,F) and frontonasal processes (Figure 7 D,H) of stage-matched *RockDN* embryos, but was found in the same pattern as the controls in the neural ectoderm, which is not derived from NCC (Figure 7 G,H). Moreover, there were foci of intense phalloidin staining scattered throughout the ectomesenchyme of the pharyngeal arches and frontonasal process of mutant embryos, which were rarely seen in control embryos (blue arrow in Figure 7 F,H). These foci of rounded up, intensely phalloidin-stained cells were interspersed with activated caspase 3-expressing cells (white arrow in Figure 7 F,H), with occasional phalloidin-intense cells also expressing activated caspase 3 (arrowheads in Figure 7 F,H). Thus, disruption of the actin cytoskeleton is associated with cell death in the craniofacial region.

**Figure 8:**
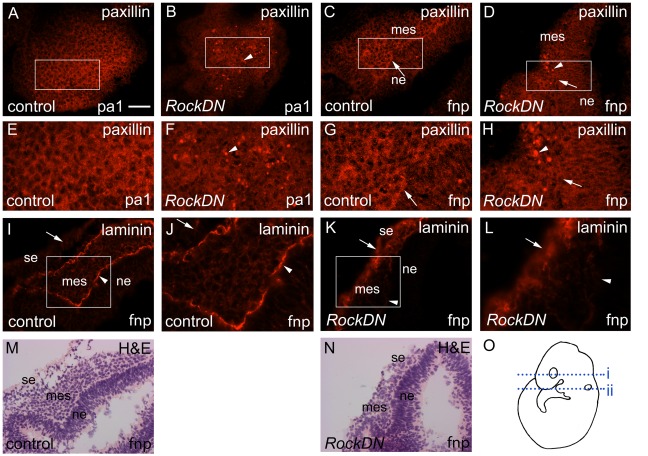
Disruption of focal adhesions and extracellular matrix in E9.5 *RockDN;Wnt1-cre* embryos. A,B,E,F are sections of the first pharyngeal arch (line i in O) and C,D,G,H,I–N are from the frontonasal processes (line ii in O). **A–H**) E–H are magnified regions shown in the boxes on A–D, respectively. Paxillin has a cortical distribution in the first pharyngeal arch in the control embryos (A,E) and also marks the boundary between the neural ectoderm and NCC-derived ectomesenchyme (arrow in C,G) in the frontonasal processes. This boundary staining is lost in the mutant (arrow in D,H) and there are intense paxillin positive foci in the pharyngeal arch and frontonasal processes (arrowheads in B,F,D,H), confirming loss of cell-substrate adhesion. **I–L**) Laminin was lost from the ectomesenchyme-neural ectoderm boundary (compare arrowhead in J with L) and was abnormally distributed in the surface ectoderm in the frontonasal processes in the *RockDN;Wnt1-cre* embryos (compare arrow in I with K). The H&E staining of the same sections are shown in M and N, allowing visualisation of the different cellular layers. fnp = frontonasal process; pa1 = first pharyngeal arch; ne = neural ectoderm; se = surface ectoderm; mes = mesenchyme. Scale bar in A–D,I,K = 50 µm; M,N = 40 µm.

In cell culture, vinculin localises to integrin-mediated focal adhesion complexes, linking the internal cytoskeleton of the cell to the substrate on which it sits, and also, at lower levels, in cadherin-mediated cell-cell junctions (reviewed in [20]). As detachment of cells from the matrix has been observed in *RockDN*-expressing motor neurones [12], we looked for evidence of disruption of cell-matrix adhesion, as a possible cause of the cell death we observe in the craniofacial region of *RockDN* mutants. Whereas in cultured NCC, vinculin localised to distinct foci at the periphery of the cells as expected (data not shown), this was not the case in the three dimensional environment of the developing embryo. In control embryos, vinculin was expressed strongly in discrete regions of the tissue, associated with boundaries between different cell types, such as the boundary between the NCC-derived ectomesenchyme and the neural ectoderm (Figure 7 K,O). Lower levels were also found at the periphery of individual cells, likely reflecting its association with cadherin-based cell-cell junctions. In matched *RockDN* embryos, vinculin was lost at the ectomesenchyme : neural ectoderm boundary but was expressed more broadly in the NCC-derived ectomesenchyme, in both the first pharyngeal arch (Figure 7 I,J,M,N) and in the frontonasal region (Figure 7 K,L,O,P). Ectopic vinculin expression was also found in the surface ectoderm of the frontonasal region and pharyngeal arches (Figure 7 J,L). Co-staining for vinculin and activated-caspase 3 showed some localisation within the same areas, suggesting a possible link between disruption of the actin cytoskeleton, cell-substrate adhesion and cell death of the *RockDN*-expressing NCC. Staining for paxillin, another component of focal adhesions, confirmed these observations (Figure 8 A–H). Paxillin was localised to the ectomesenchyme : neural ectoderm boundary and individual cell membranes in control embryos (Figure 8 A,C). However, staining was lost from the ectomesenchyme : neural ectoderm boundary in *RockDN* embryos and was observed in punctate foci in the ectomesenchyme (Figure 8 F,H) in a similar distribution to activated-caspase 3 immunoreactivity, supporting the idea that cell death was occurring at sites of abnormal focal adhesion formation. Immuno-staining for laminin confirmed that the interactions with the extracellular matrix were abnormal, as basal lamina localisation was lost from the ectomesenchyme : neural ectoderm boundary in the frontonasal processes and was abnormally distributed throughout the surface ectoderm in both the frontonasal processes and the pharyngeal arches (Figure 8 I–L and data not shown).

## Discussion

We have shown a cell-autonomous requirement for Rock function within NCC in the development of the frontal aspect of the face, and that in the absence of this, there is increased cell death within the forming facial protuberances leading to hypoplasia and midline facial clefting. Cell-extracellular matrix adhesion, and specifically the distribution of focal adhesion proteins and the basement membrane protein laminin, is abnormal in the ectomesenchyme from *RockDN* mutants. Rock is known to play important roles in modulating the cytoskeleton [4], [5] and the induction of stress fibres by the RhoA-Rock pathway is required for the formation of focal adhesions [21]. Thus, the cell death resulting from Rock inhibition is likely to be a secondary consequence of cytoskeletal disorganisation, as the NCC lose adhesion to the surrounding extracellular matrix, round up, and die. Similarly, reduced neuronal survival and apoptotic cell death are seen when Rock function is abrogated in developing motor neurons [12]. These authors suggested that anoikis, cell death as a result of failing to interact with the surrounding matrix, occurs in the absence of Rock; we also favour this as the mechanism leading to high levels of cell death in the *RockDN;Wnt1-cre* embryos described here.

Although the role of focal adhesions has been well explored in the two-dimensional environment of the culture dish, little is known about the role of focal adhesions within living tissues. In cells in three-dimensional matrices, focal adhesions cannot be detected as punctate aggregates at the cell surface [22]. In the developing craniofacial region of normal mouse embryos, we mainly detected vinculin localised to the boundaries between different cell types, for example the region between the ectomesenchyme and ectoderm of the developing frontonasal process. These boundaries are the sites of basement membranes, which are specialisations of the extracellular matrix that provide structural support for the surrounding tissues, and where cell-adhesion to the matrix is crucial [23]. The basement membrane component laminin, was found in a similar distribution to vinculin at these tissue boundaries in the craniofacial region of control embryos. Laminin and vinculin were lost from the ectomesenchyme : neural ectoderm boundary in the frontonasal processes, but were markedly elevated in the surface ectoderm of both this region and the pharyngeal arches. Thus, the relationship between the ectomesenchyme and neighbouring tissue types appears to be disrupted. This potential role for Rock1 at the basement membrane is supported by a recent publication [24] that has shown that Rock1 regulates basement membrane placement at the basal surface of the epithelium of the developing salivary gland, playing a role in establishing polarity within the tissue. In this scenario, Rock inhibition resulted in accumulation of basement membrane proteins throughout the epithelium [24]. Similarly, we see excessive vinculin and laminin within the surface ectoderm, but also within the ectomesenchyme, of the pharyngeal arches and the frontonasal processes, suggesting Rock1 may be playing a similar roles in restricting basement membrane to the basal surface of the epithelium in the developing craniofacial region. The knockdown of Rock only in the ectomesenchyme (NCC) in our mutant, suggests that this effect can be non-cell autonomous.

Both phalloidin, which binds filamentous actin, and paxillin, another component of focal adhesions, were found in condensed foci in the ectomesenchyme of *RockDN* mutants, which were intermingled with dying cells. Thus, increased cell death was associated with disruption of the cytoskeleton and abnormal distribution of focal-adhesion proteins. The observation that only a small number of phalloidin/paxillin-rich condensed foci co-expressed activated-caspase 3 likely relates to the role the latter plays in the process of cell death, regulating chromatin condensation and DNA fragmentation [25]. Thus, activated-caspase 3 would only be expected in the ectomesenchymal cells in the latter stages of cell death. We propose that in contrast, the disruption of focal adhesion and cytoskeletal proteins, and the consequent detachment from the surrounding matrix, is the cause of the cell death, and thus would be apparent within the tissue for much longer. Rac1 has also been implicated in playing an essential role in regulating cell-matrix adhesion in NCC [26] and *Rac1^f/f^;Wnt1-cre* embryos develop similar craniofacial abnormalities as the *RockDN;Wnt1-cre* embryos. Excessive cell death, in combination with disrupted NCC-matrix adhesion was observed in the in the frontonasal region at E11.5 in these embryos, once the midfacial clefting was apparent, although the authors did not look earlier, before the defects arose. Together, these data suggest that it is the defect in the interaction of NCC with the surrounding matrix, with the multiple effects that this has on the tissue, that is the crucial factor in the development of the craniofacial malformations observed.

Frontonasal dysplasia has been described as a consequence of loss of expression of certain transcription factors, including *Alx3* and *Alx4*. Although alone, *Alx3* null mice have no phenotype, severe midfacial clefting and truncation of the snout is observed when the mice are inter crossed with *Alx4* null mice (*Alx3^+/−^;Alx4^−/−^* and *Alx3^−/−^;Alx4^−/−^;* [27]). Excessive cell death was observed in the frontonasal processes at E10.0, before the appearance of the defects, as we have observed in *RockDN;Wnt1-cre* mice. Similarly, embryos null for the related transcription factor *Cart1*, when combined with loss of *Alx4 (Alx4^−/−^;Cart1^+/−^* and *Alx4^−/−^;Cart1^−/−^*), also display a similar phenotype, with severe midfacial clefting and open eyes at birth [28], as we see in the *RockDN;Wnt1-cre* embryos. Brachyrrhine (*3H1 Br/+*) mice display frontonasal dysplasia, thought to be caused by loss of the developmentally important transcription factor *Six2* [29]–[31], although in this case associated with reduced proliferation in the craniofacial region, rather than increased cell death [30]. Nevertheless, the consequences of reduced proliferation and excessive cell death in a particular tissue are similar, with an overall reduction in cell numbers relative to normal controls. Thus, a reduction in the numbers of NCC within the developing midface appears to be the critical factor for the development of frontonasal dysplasia.

### Conclusions

Rodent models of craniofacial abnormalities represent a valuable tool for studying, understanding and ultimately preventing human malformations. For example, mutations in *ALX3*, whose mouse counterpart has been implicated in the development of the midface [27] also cause frontorhiny, a form of midfacial clefting in humans [32]. Our study suggests an important role for Rock and other regulators of the cytoskeleton in maintaining normal craniofacial morphogenesis. In humans, frontonasal dysplasia (OMIM: 136760) is considered to be a sporadic event, supporting the idea that craniofacial malformations have a complex aetiology. Although there are no reports of mutations in *ROCK1* or *ROCK2* in patients with craniofacial malformations, loss of both chromosome 18q and 2p (where *ROCK1* and *ROCK2* reside, respectively) result in a spectrum of abnormalities that include hypertelorism, palatal defects and micrognathia and cardiac (OMIM 218340 and 601808). Thus, haploinsufficiency for these genes might contribute to the phenotype of these patients. From our data, and a review of the current literature relating to frontonasal dysplasia and midface clefting resulting from mutations in a range of different genes, we suggest that the common link is a reduction in NCC in the developing frontonasal prominences and first pharyngeal arch, in many cases related to increased NCC apoptosis. The key roles that Rho kinase plays in regulating the cytoskeleton and maintaining cell-matrix interactions suggests that it may be a common downstream effector in this cell death process.

## Materials and Methods

### Ethics Statement

Ethical approval of animal work carried out in this project has been authorised by the Newcastle University Ethics Committee and was covered by Project Licence PPL 60/3876 approved by the UK Home Office.

### Mice and embryos

Rock dominant-negative (*RockDN*) mice [12] (BRC no. 01294) from RIKEN BioResource Center (Tsukuba, Japan) were inter-crossed with the *Rosa 26R* reporter line [33], *ROSA-EYFP* line [34], *Wnt1-cre* line [13] or the PGK-cre line [16]. Mice were maintained according to the Animals (Scientific Procedures) Act 1986, United Kingdom. Genotyping for *RockDN* positive mice was performed as described [12]. CD1 mice were obtained from Charles River. Stage matched mutant and control embryos were used for all experiments.

### Quantitative real-time PCR

The relative quantities of the *CAT* gene cassette was measured by quantitative real time PCR. RNA was extracted from four E11.5 control and four mutant pharyngeal arches 1 and 2 using the Trizol reagent kit (Invitrogen) in duplicate. cDNA was produced using 1 µg of RNA with an initial DNase treatment step (Invitrogen DNase kit) followed by reverse transcription (Invitrogen SuperScript II RTase kit). Random 15-mers were used (Sigma).

Quantitative real-time PCR was performed using a 7900ht fast real-time PCR system (Applied Biosystems) and the SYBR green JumpStart Taq ReadyMix kit (Sigma). All reactions were performed in triplicate. Two housekeeping genes were used for normalisation, *β-actin* and *GAPDH*, and were found to be stably expressed in this experimental setting. Relative quantities of gene expression were calculated using the ΔΔCt method. Primer sequences were for *β-actin*, F: 5′-GCTGGTCGTCGACAACGGCTC-3′, R: 5′-CAAACATGATCTGGGTCATCTTTTC-3′, for *GAPDH* F:5′-GCTGGTCGTCGACAACGGCTC-3′, R:5′-CAAACATGATCTGGGTCATCTTTTC-3′ and for *CAT* primers see [12].

### Head Measurements

Heads were collected from E15.5 embryos, digital protographs were taken, and measurements were made using ImageJ software [35].

### Histology and skeletal staining

β-gal staining of *RockDN;Rosa 26R* embryos was performed as described previously [36]. For skeletal staining, the superficial muscle layers, eyes and internal organs were removed before fixing in Bouin's solution. E18.5 embryos were dehydrated in 95% ethanol and washed in acetone before staining for cartilage in alcian blue for eight days. Bone was stained with alizarin red. E14.5 embryos were washed with 70% ethanol/0.1% NH_4_OH and cartilage stained in 0.05% alcian blue/5% acetic acid. Embryos were imaged in either glycerol or benzyl alcohol/benzylbenzoate.

### Immunohistochemistry

Immunolabelling was performed on PFA-fixed, paraffin-embedded sections using the Rock2 antibody (Santa Cruz Biotechnology). Cryosections were labelled using antibodies raised against activated Rock1 (abcam), caspase 3 (Cell Signalling), vinculin (Sigma), paxillin (abcam), laminin (Sigma), GFP-FITC (Molecular Probes) and filamentous actin was stained with rhodamine-phalloidin (Sigma). Each experiment was repeated a minimum of three times and included appropriate controls.

The mean percentage cell death and cell proliferation was calculated from three matched sections of the first pharyngeal arch from four control and four mutant E9.5 embryos.

### Statistical analysis

Statistical analysis was carried out using SPSS (IBM). The one way ANOVA test and one sample t-test were used as appropriate.

## Supporting Information

Figure S1 **Craniofacial phenotype in** ***RockDN;Wnt1-cre*** **embryos.** Neural crest cells and their derivatives are stained blue in each case. **A,B**) The frontonasal region is hypoplastic in *RockDN* embryos at E9.5. **C–F**) Clefting of the midface is obvious in *RockDN* embryos at E11.5 and E13.5, with wide separation of the lateral prominences (white lines in D,F). There is also marked hypertelorism (double arrows in E,F). **G–J**) In some mildly affected mutants, the facial clefting in *RockDN* mutants is apparent only as a midline cleft lip and a bifid nasal tip (white arrows in H,J). Hyperteleorism is still apparent in these more mildly affected embryos however (double headed arrows in G,H; arrows are same length in each case). I and J are magnified images of the frontonasal region from embryos in G and H respectively. Scale bar in A–D = 200 µm; E,F = 330 µm; G,H = 400 µm.
(TIF)

Figure S2 **Expression of Rock1 in the pharyngeal arch and Rock2 in the developing craniofacial region at E10.5.** **A–D**) Rock1 (red) is expressed throughout the pharyngeal arch at E9.5 (A,B) and E10.5 (C,D). **E–G**) Rock2 protein is expressed in and around the dorsal root ganglia, adjacent to the neural tube (E), within pharyngeal arch 1 (F), and within the ectomesenchyme of the frontonasal processes (G). fnp = frontonasal process; nt = neural tube; pa1 = pharyngeal arch 1. Scale bar A–E = 50 µm.
(TIF)

Figure S3 **NCC in the frontonasal region at E9.5 in** ***RockDN;Wnt1-cre*** **embryos.** **A,B**) The intensity of NCC (blue) in the frontonasal processes (arrow) is reduced in the *RockDN;Wnt1-cre* mutant embryo (B) compared to the wildtype littermate (A). **C**) Shows the position of the section shown on an E9.5 embryo. e = eye. Scale bar = 50 µm.
(TIF)
