# Supplementary figures and images for: Neural Crest Cell Survival Is Dependent on Rho Kinase and Is Required for Development of the Mid Face in Mouse Embryos
Source: PLoS One. 2012 May 21;7(5):e37685. doi: 10.1371/journal.pone.0037685 (PMC3357402; doi:10.1371/journal.pone.0037685)

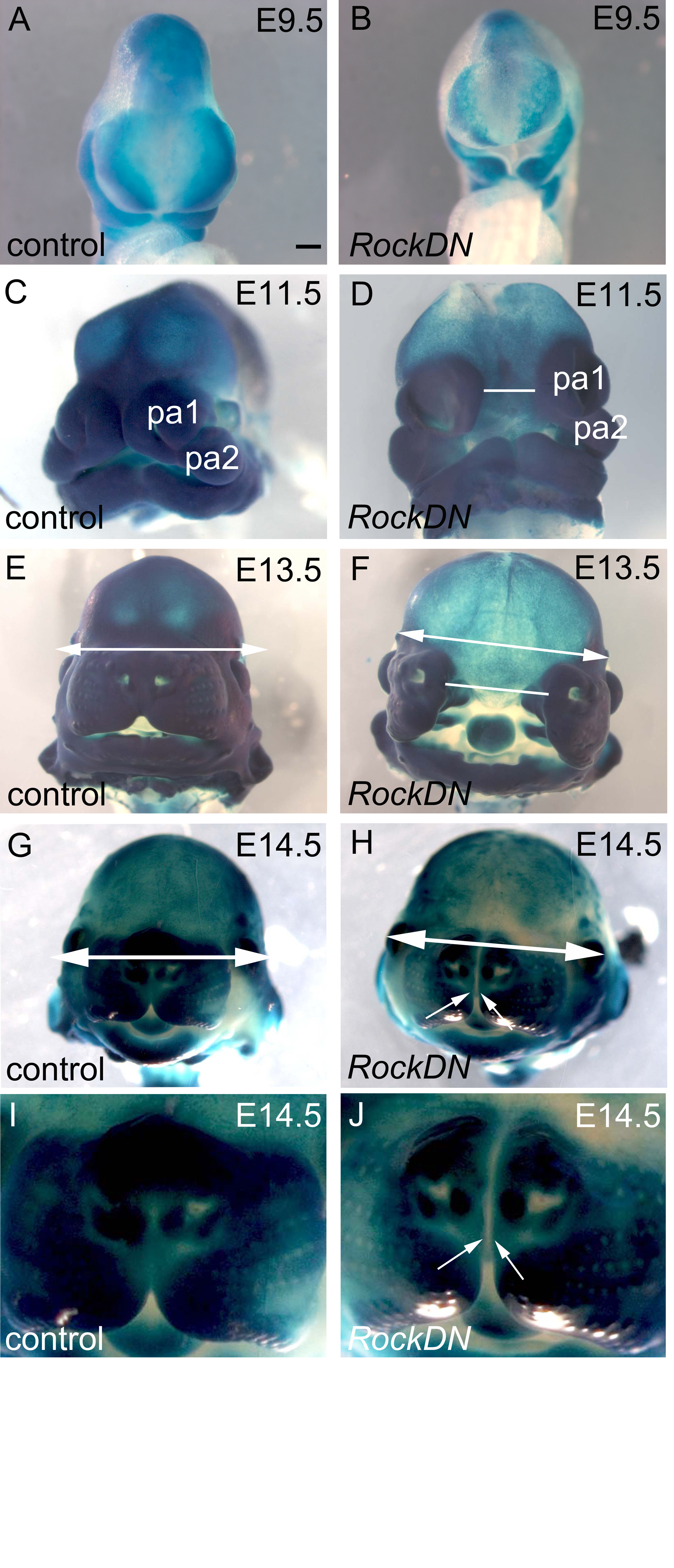

Supplement: Figure S1 — Craniofacial phenotype in RockDN;Wnt1-cre embryos. Neural crest cells and their derivatives are stained blue in each case. A,B) The frontonasal region is hypoplastic in RockDN embryos at E9.5. C–F) Clefting of the midface is obvious in RockDN embryos at E11.5 and E13.5, with wide separation of the lateral prominences (white lines in D,F). There is also marked hypertelorism (double arrows in E,F). G–J) In some mildly affected mutants, the facial clefting in RockDN mutants is apparent only as a midline cleft lip and a bifid nasal tip (white arrows in H,J). Hyperteleorism is still apparent in these more mildly affected embryos however (double headed arrows in G,H; arrows are same length in each case). I and J are magnified images of the frontonasal region from embryos in G and H respectively. Scale bar in A–D = 200 µm; E,F = 330 µm; G,H = 400 µm. (TIF) [file pone.0037685.s001.tif]

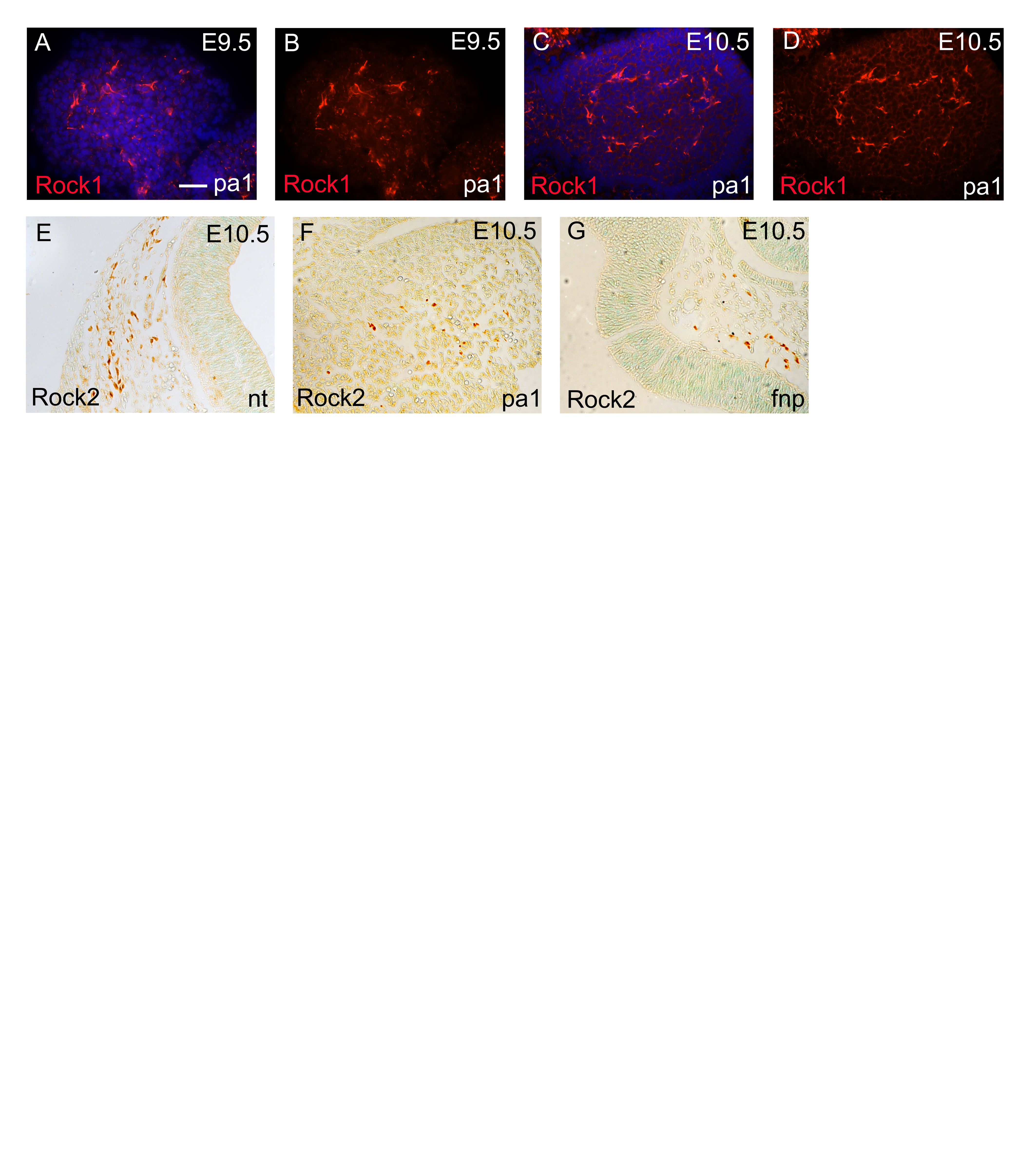

Supplement: Figure S2 — Expression of Rock1 in the pharyngeal arch and Rock2 in the developing craniofacial region at E10.5. A–D) Rock1 (red) is expressed throughout the pharyngeal arch at E9.5 (A,B) and E10.5 (C,D). E–G) Rock2 protein is expressed in and around the dorsal root ganglia, adjacent to the neural tube (E), within pharyngeal arch 1 (F), and within the ectomesenchyme of the frontonasal processes (G). fnp = frontonasal process; nt = neural tube; pa1 = pharyngeal arch 1. Scale bar A–E = 50 µm. (TIF) [file pone.0037685.s002.tif]

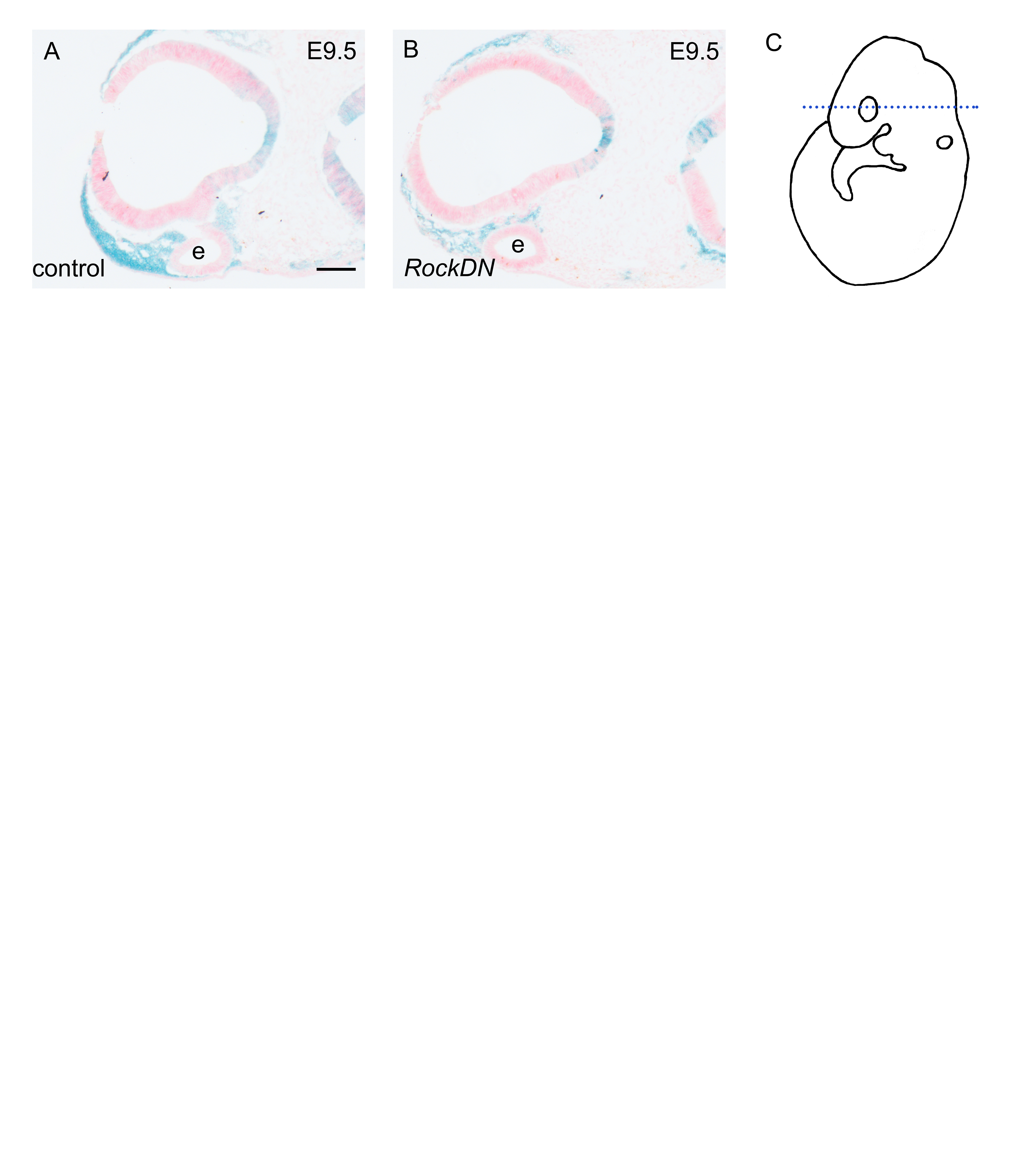

Supplement: Figure S3 — NCC in the frontonasal region at E9.5 in RockDN;Wnt1-cre embryos. A,B) The intensity of NCC (blue) in the frontonasal processes (arrow) is reduced in the RockDN;Wnt1-cre mutant embryo (B) compared to the wildtype littermate (A). C) Shows the position of the section shown on an E9.5 embryo. e = eye. Scale bar = 50 µm. (TIF) [file pone.0037685.s003.tif]
